# Linkages and Interactions Analysis of Major Effect Drought Grain Yield QTLs in Rice

**DOI:** 10.1371/journal.pone.0151532

**Published:** 2016-03-28

**Authors:** Prashant Vikram, B. P. Mallikarjuna Swamy, Shalabh Dixit, Jennylyn Trinidad, Ma Teresa Sta Cruz, Paul C. Maturan, Modesto Amante, Arvind Kumar

**Affiliations:** Plant Breeding Genetics and Biotechnology Division, International Rice Research Institute (IRRI), Los Baños, Metro Manila, Philippines; National Institute of Plant Genome Research, INDIA

## Abstract

Quantitative trait loci conferring high grain yield under drought in rice are important genomic resources for climate resilient breeding. Major and consistent drought grain yield QTLs usually co-locate with flowering and/or plant height QTLs, which could be due to either linkage or pleiotropy. Five mapping populations used for the identification of major and consistent drought grain yield QTLs underwent multiple-trait, multiple-interval mapping test (MT-MIM) to estimate the significance of pleiotropy effects. Results indicated towards possible linkages between the drought grain yield QTLs with co-locating flowering and/or plant height QTLs. Linkages of days to flowering and plant height were eliminated through a marker-assisted breeding approach. Drought grain yield QTLs also showed interaction effects with flowering QTLs. Drought responsiveness of the flowering locus on chromosome 3 (*qDTY*_*3*.*2*_) has been revealed through allelic analysis. Considering linkage and interaction effects associated with drought QTLs, a comprehensive marker-assisted breeding strategy was followed to develop rice genotypes with improved grain yield under drought stress.

## Introduction

Genetic linkages and interactions are the two most important aspects accounting for the complexity of quantitative traits such as drought tolerance in rice. Such issues could not be efficiently addressed through a classical Mendelian approach [1]. However, recent advances in molecular marker applications have enabled plant breeders to better understand the genetic basis of complex traits [2,3,4,5]. The genetic basis of drought tolerance in rice, a model crop species, has been examined through mapping and fine mapping of QTLs. Major-effect drought grain yield QTLs have now been identified, but the use of tightly linked markers is still a limitation because of their co-location with other loci that may be undesirable for a plant breeder. For example, the major and consistent drought grain yield (GY) QTLs that can significantly enhance grain yield under reproductive-stage drought stress (RS) usually coincide with QTLs for plant height (PH) and/or flowering (DTF). The most consistent drought GY QTL identified so far, *qDTY*_*1*.*1*_, co-located with QTLs related to PH and DTF under stress. Interestingly, *qDTY*_*1*.*1*_ harbours green revolution gene '*sd1*', and it has long been debated whether linkages or pleiotropic effects are associated with this gene [6,7,8,9,10]. Positive allele of *qDTY*_*1*.*1*_ has been reported by upland adapted traditional cultivars N22 and Dhagaddesi [9,11]. Another major QTL, *qDTY*_*3*.*1*_, identified for lowland drought stress co-located with QTLs for DTF. This QTL was also contributed by an upland adapted variety ‘Apo’ [12]. The largest effect drought GY QTL reported to date, *qDTY*_*12*.*1*_, also co-located with QTLs for DTF, PH, and some other drought-related traits such as biomass, harvest index, panicle number, and drought response index [13]. *qDTY*_*12*.*1*_ was identified in Vandana/Way Rarem population, both parents are upland adapted cultivars, positive allele being contributed by Way Rarem. Similar to the drought GY loci, other drought QTLs also co-locate with the regions governing different drought-related traits. *qDTY*_*3*.*2*_ is one such example. This locus, known as ‘*HD9*,’ was first identified as a major flowering locus [14] later on DTF, PH and some other drought related traits positive allele being contributed from Vandana [13]. This QTL is recently reported to be associated with GY under drought [9] and markers underlying this QTL region were reported to interact with drought GY QTLs [15]. Characterization of *qDTY*_*3*.*2*_ is therefore important to understand its genetic and genomic basis for practical breeding applications.

From a plant breeder’s perspective transfer of a DTH/PH loci collocating with drought GY loci may not be a preferred phenomenon and is a common phenomenon due positive allele contribution from tall and early donor genotypes. As an example, for rain-fed lowland situation medium to long duration variety with semi-dwarf height is preferred apart from its tolerance to drought. Co-location of the drought-related QTLs could be for three possible reasons: genetic linkage, pleiotropic effects, and cause-and-effect association [16]. From among these three possibilities, linkages between genes for individual traits or pleiotropic effects of genes affecting multiple traits are more commonly supported [17,18]. Recently, efforts have been made to characterize these co-locations for some rice drought GY QTLs. Covariate analysis has been carried out to remove the confounding effect of DTF on GY under drought [9,12].

Main and interaction effects of QTLs are usually estimated through composite interval mapping (CIM) models that consider a single trait at a time, but this approach does not allow an evaluation of pleiotropic effects. Multiple-trait mapping methods offer a powerful alternative approach for testing pleiotropic effects of the QTLs and therefore help in increasing the accuracy of mapping correlated traits [19]. Distinguishing linkage from pleiotropy is considered to be a difficult task requiring a large population of segregating individuals, greater marker saturation in the QTL region, and suitable multiple-trait analysis methods [19]. Despite these challenges, testing genetic linkage versus pleiotropy is recommended prior to QTL application in marker-assisted breeding (MAB). Considering these challenges, a suitable MAB strategy for drought tolerance in rice needs to be defined. In view of this the objectives of our study were framed which include- (1) testing the significance of pleiotropism for three major drought GY QTLs (*qDTY*_*1*.*1*_, *qDTY*_*3*.*1*_, and *qDTY*_*12*.*1*_); (2) validation of the *qDTY*_*3*.*2*_ effect on DTF and GY under RS and analysis of its interaction effects with other QTLs for GY under drought (3) marker-assisted linkage elimination from the drought GY QTLs- *qDTY*_*1*.*1*_, *qDTY*_*3*.*1*_, and *qDTY*_*12*.*1*_.

## Results

### Test of pleiotropism of the drought GY QTLs through multiple-trait mapping

MT-MIM was carried out for three QTLs in five populations to determine the LOD of the pleiotropic effects of *qDTY*_*1*.*1*_, *qDTY*_*3*.*1*_, and *qDTY*_*12*.*1*_. GY QTLs were analyzed in pairs with DTF/PH QTLs for each of the three regions and LOD, and the significance of the pleiotropic effects was determined. The LOD of pleiotropic effects for the GY-PH QTL pair in *qDTY*_*1*.*1*_ ranged from 0.7 to 2.4 and from 1.3 to 2.1 for the GY-DTF QTL pair in the same region. None of the LOD values for GY-PH and GY-DTF for different QTLs were significant (Table 1).

**Table 1:** Multiple-trait, multiple-interval mapping (MT-MIM) to test the pleiotropic effect of drought grain yield QTLs on flowering and plant height.

QTL name	Population	Marker interval	Trait 1	Trait 2	Season	LOD of pleiotropy effect	LOD threshold
*qDTY*_*1*.*1*_	N22/Swarna	RM11943—RM12091	GY	DTF	Year 1	1.4	2.4
					Year 2	1.5	2.1
			GY	PHT	Year 1	1.7	2.3
					Year 2	0.7	2.1
	N22/IR64	RM11943—RM12091	GY	DTF	Year 1	1.3	2.5
					Year 2	2.1	2.4
			GY	PHT	Year 1	2.4	2.7
					Year 2	0.7	2.2
*qDTY*_*3*.*1*_	Apo/2*Swarna	RM520—RM16030	GY	DTF	Year 1	1.3	2.7
					Year 2	1.9	3.1
			GY	PHT	Year 1	1.1	2.6
					Year 2	0.7	2.5
*qDTY*_*12*.*1*_	Vandana/Way Rarem	RM28048—RM511	GY	DTF	Year 1	0.7	4.2
					Year 2	1.2	13.3
			GY	PHT	Year 1	1.5	3.3
					Year 2	2.6	9.3
	IR74371-46-1-1/2*Sabitri	RM28166—RM28199	GY	DTF	Year 1	1.7	6.2
					Year 2	0.6	13.2
			GY	PHT	Year 1	0.4	3.9
					Year 2	0.6	12.6

LOD threshold was significant at 1%.

### Characterization of the flowering locus for co-localized drought related traits

**Fig 1:**
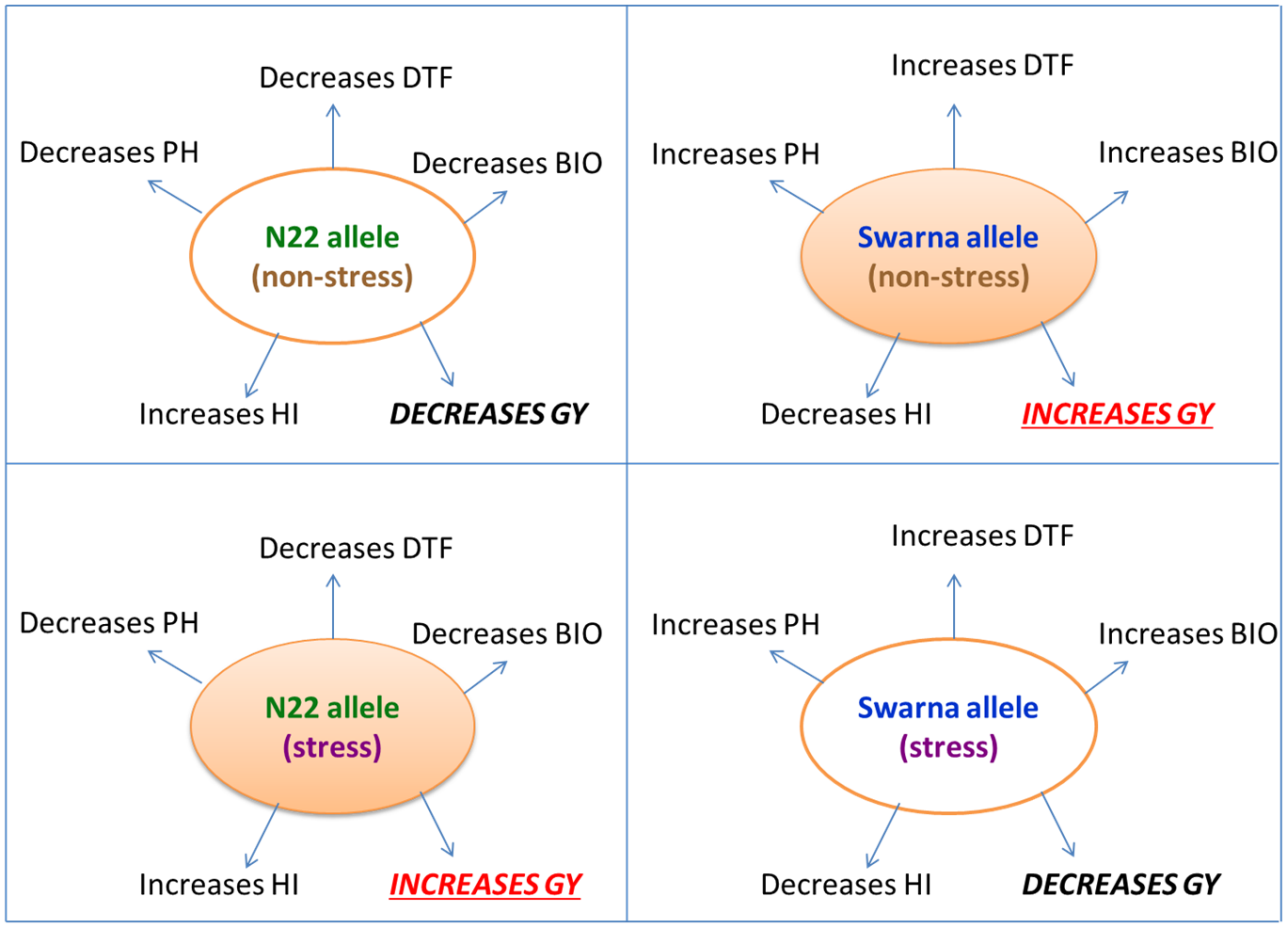
Figure representing the effect of N22 and Swarna alleles at *qDTY*_*3*.*2*_ locus. GY increase under RS due to N22 allele and under NS due to Swarna allele confirms the drought responsiveness of the *qDTY*_*3*.*2*_ locus.

The flowering locus *qDTY*_*3*.*2*_ showed significant effects for GY and DTF in the N22/Swarna BIL population under reproductive-stage drought stress (RS) drought and non-stress (NS) situations of two seasons, DS2011 and DS2012. QTL effects have been presented as additive effects percentage, a measure which is more practical compared to other measures. This was estimated as the percentage of population mean contributed by QTL [Additive effect/population mean × 100]. Additive effects were contributed by N22 and Swarna (denoted as ‘-’ sign in Table 2). Additive effects, F-values and allele contributions have been presented in a separate column in Table 2. Increases in DTF, PH, and BIO under RS and NS were contributed by Swarna, whereas N22 contributed the HI-increasing allele under RS as well as NS for *qDTY*_*3*.*2*_. Interestingly, enhancement of GY under RS came from N22, whereas it came from Swarna under NS, indicating the drought responsiveness of *qDTY*_*3*.*2*_. The effect of the N22 and Swarna alleles in the *qDTY*_*3*.*2*_ region was similar in the N22/Swarna BIL and N22/Swarna RIL populations (Table 2, Fig 1).

**Table 2:** Effect of *qDTY*_*3*.*2*_ for high grain yield under drought in N22/Swarna BIL and RIL populations.

	F-value				Additive effect %				Allele contribution			
Season	Season1		Season2		Season1		Season2		Season1		Season2	
Environment	Non-stress	Stress	Non-stress	Stress	Non-stress	Stress	Non-stress	Stress	Non-stress	Stress	Non-stress	Stress
N22/Swarna BIL population												
GY	13.7	27.1	29.8	42.6	-17.1	23.9	-22.8	49.2	Swarna	N22	Swarna	N22
DTF	60.7	85.1	62.8	52.6	-14.8	-15.6	-8.9	-10.1	Swarna	Swarna	Swarna	Swarna
PH	26.9	46.7	14.5	5.2	-5.3	-5.8	-4.3	-2.9	Swarna	Swarna	Swarna	Swarna
BIO	18.9	4.6	^NA^	^NA^	-14.1	-9.1	^NA^	^NA^	Swarna	Swarna	^NA^	^NA^
HI	5.7	42.8	^NA^	^NA^	7.7	35.3	^NA^	^NA^	N22	N22	^NA^	^NA^
N22/Swarna RIL population												
GY	3.70 ^NS^	3.16 ^NS^	7.8	17.1	-3.4	3.4	-6.8	13.6	Swarna	N22	Swarna	N22
DTF	125.5	135.5	113.2	79.6	-5.3	-8	-5.6	-4	Swarna	Swarna	Swarna	Swarna
PH	12.5	6.7 ^NS^	10.9	0.5^NS^	-3.9	-1.1	-3.5	-1.9	Swarna	Swarna	Swarna	Swarna
BIO	58.7	24.6	39.3	0.8 ^NS^	-20.5	-10.2	-12	-0.9	Swarna	Swarna	Swarna	Swarna
HI	36.4	13.5	8.2	73.6	10.7	11.1	3.8	19.9	N22	N22	N22	N22

^NA^: not available, BIO and HI were recorded in only one season in N22/Swarna BIL population.

^NS^: non-significant. F-value: represent the significance of QTLs and their effects. Additive effect (%): Additive effect / Population mean × 100. Allele contribution: represents the parents contributing positive allele for effect of QTL.

### Interaction analysis of drought related grain yield and flowering loci

*qDTY*_*1*.*1*_ showed a significant epistatic interaction with *qDTY*_*3*.*2*_ for DTF in two seasons (DS2009 and DS2010) under RS in the N22/RIL population. Both loci had a main effect on DTF and there was an additive-additive interaction between the two loci (Table 3, S1 Fig). Also in a N22/Swarna BIL population additive-additive interaction between *qDTY*_*1*.*1*_ and *qDTY*_*3*.*2*_ was observed under RS in the DS2012 experiment (data not presented). Consistent results were therefore obtained in N22 derived RIL and BIL populations. *qDTY*_*3*.*2*_ also showed an additive interaction with another drought GY QTL *qDTY*_*12*.*1*_ [15]. Therefore to better understand the effect of flowering locus *qDTY*_*3*.*2*_ on GY loci *qDTY*_*1*.*1*_ and *qDTY*_*12*.*1*_ a class analysis was carried out. QTL classes, including individual QTLs (*qDTY*_*1*.*1*_, *qDTY*_*12*.*1*_, and *qDTY*_*3*.*2*_) as well as QTL combinations (*qDTY*_*1*.*1*_ + *qDTY*_*3*.*2*_ and *qDTY*_*12*.*1*_ + *qDTY*_*3*.*2*_), were analyzed for their effect on GY and DTF under RS and NS. Results have been presented in S2 and S3 Figs. Though significant differences between different classes were not observed, a common trend was seen among three classes. Trait values (DTF or GY) of QTL combination classes showed that *qDTY*_*3*.*2*_ interact with *qDTY*_*1*.*1*_ and *qDTY*_*12*.*1*_ to reduce DTF and enhance GY under RS in different populations. Similarly, under NS, lines with *qDTY*_*1*.*1*_ / *qDTY*_*12*.*1*_ and *qDTY*_*3*.*2*_ (*qDTY*_*1*.*1*_ / *qDTY*_*12*.*1*_ + *qDTY*_*3*.*2*_) had lower yield as compared to the lines with only *qDTY*_*1*.*1*_ / *qDTY*_*12*.*1*_.

**Table 3:** *qDTY*_*3*.*2*_ main and interaction effect analysis for flowering in N22/IR64 RIL population.

Season	QTL interval 1	QTL interval 2	AA	AE%	P-value
DS2009	RM11943-RM431 (*qDTY*_*1*.*1*_)	RM22-RM231 (*qDTY*_*3*.*2*_)	-0.8	-1.2	0.003
DS2010	RM11943-RM431 (*qDTY*_*1*.*1*_)	RM22-RM231 (*qDTY*_*3*.*2*_)	-1.3	-1.6	0.005

DS2009: dry season of 2009; DS2010: dry season of 2010. AA: additive-additive interaction effect of two QTLs (*qDTY*_*1*.*1*_ and *qDTY*_*3*.*2*_). AE%: additive effect as the percent of trial mean.

### Linkage elimination from drought GY QTLs

**Fig 2:**
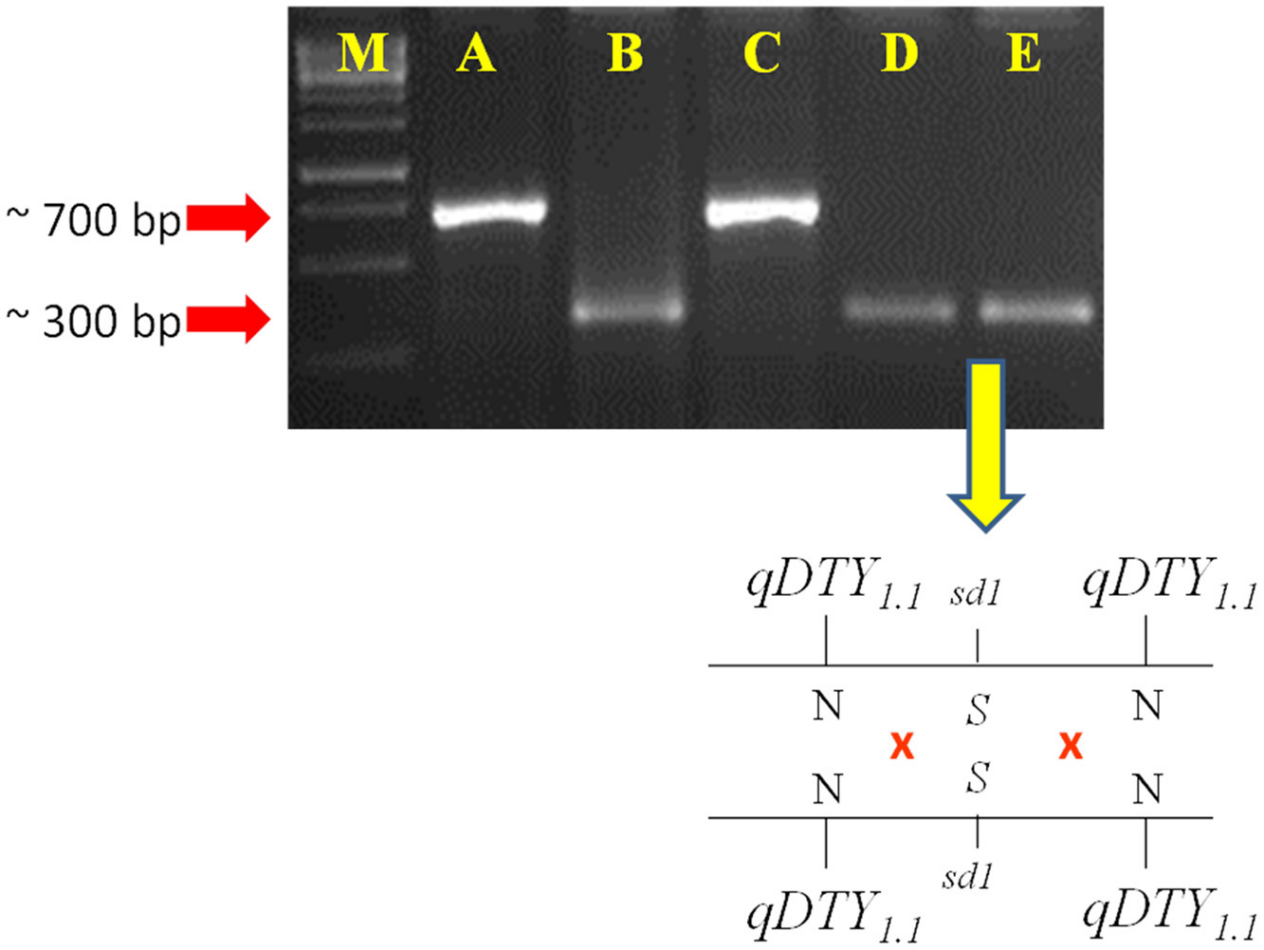
Figure representing the double cross-over event within *qDTY*_*1*.*1*_ QTL region in two N22/Swarna BILs. The semi-dwarfing gene, ‘*sd1*’ lies within *qDTY*_*1*.*1*_ region. BILs had dwarfing allele of the sd1 gene whereas N22 alleles on its both sides. M = 1Kb Ladder, A = N22, B = Swarna, C = IR 91659:25-50-B (Tall but drought susceptible N22/Swarna BIL), D = IR91659:41-59-B and E = IR91659:54-17-B (Dwarf drought tolerant *qDTY*_*1*.*1*_ N22/Swarna BILs).

**Fig 3:**
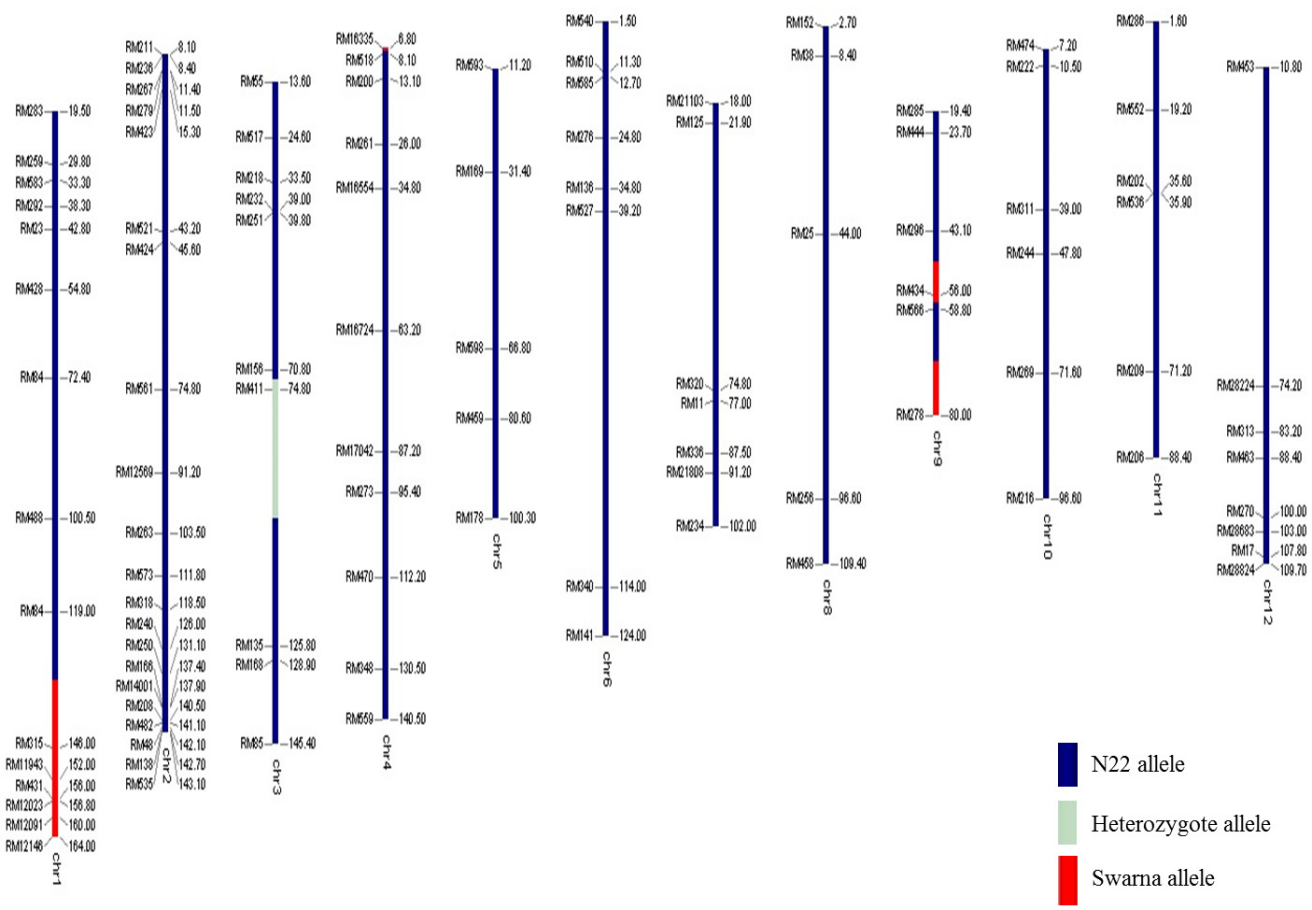
Genotypic background of *qDTY*_*1*.*1*_ BILs.

**Fig 4:**
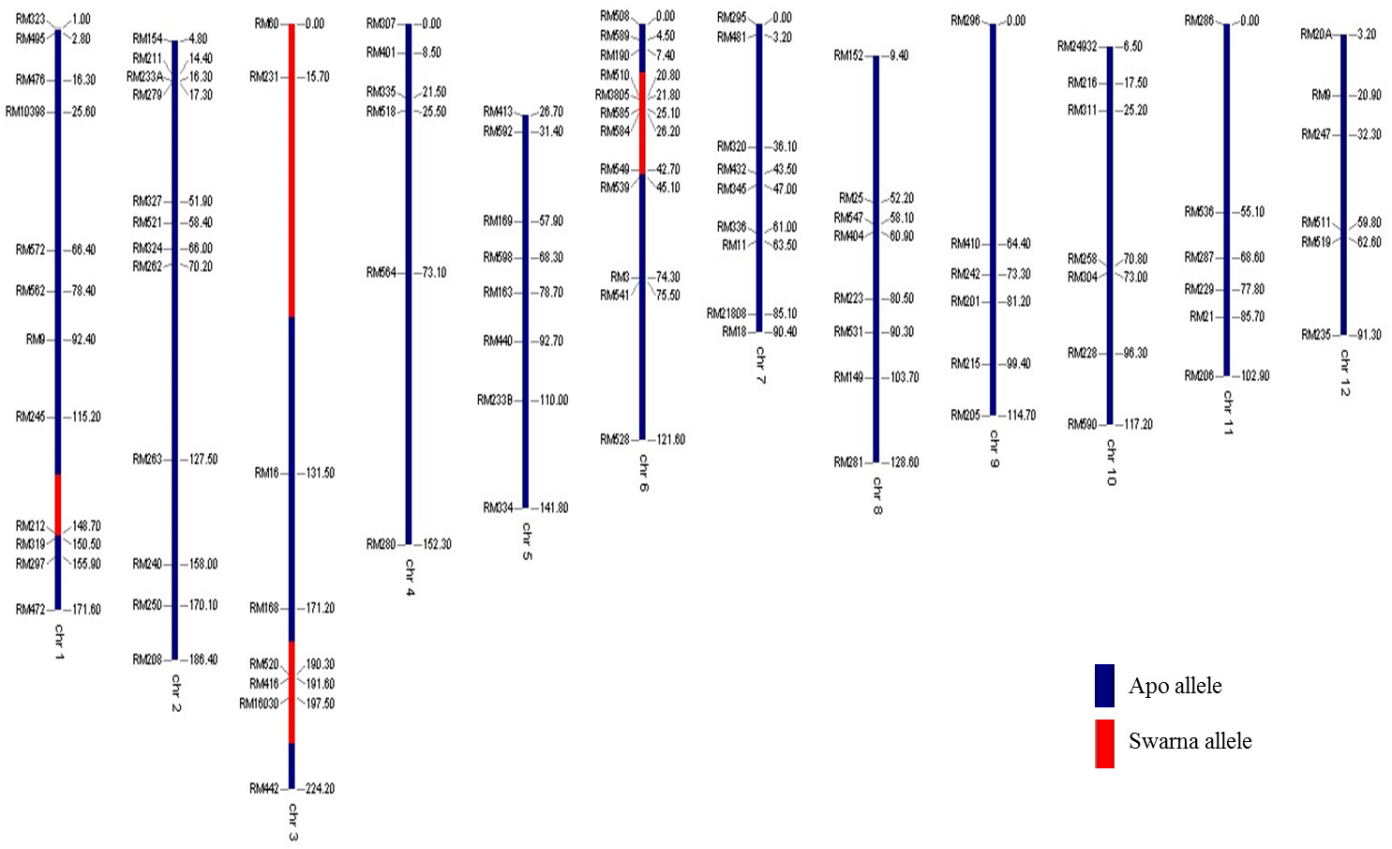
Genotypic background of *qDTY*_*3*.*1*_ BILs.

**Fig 5:**
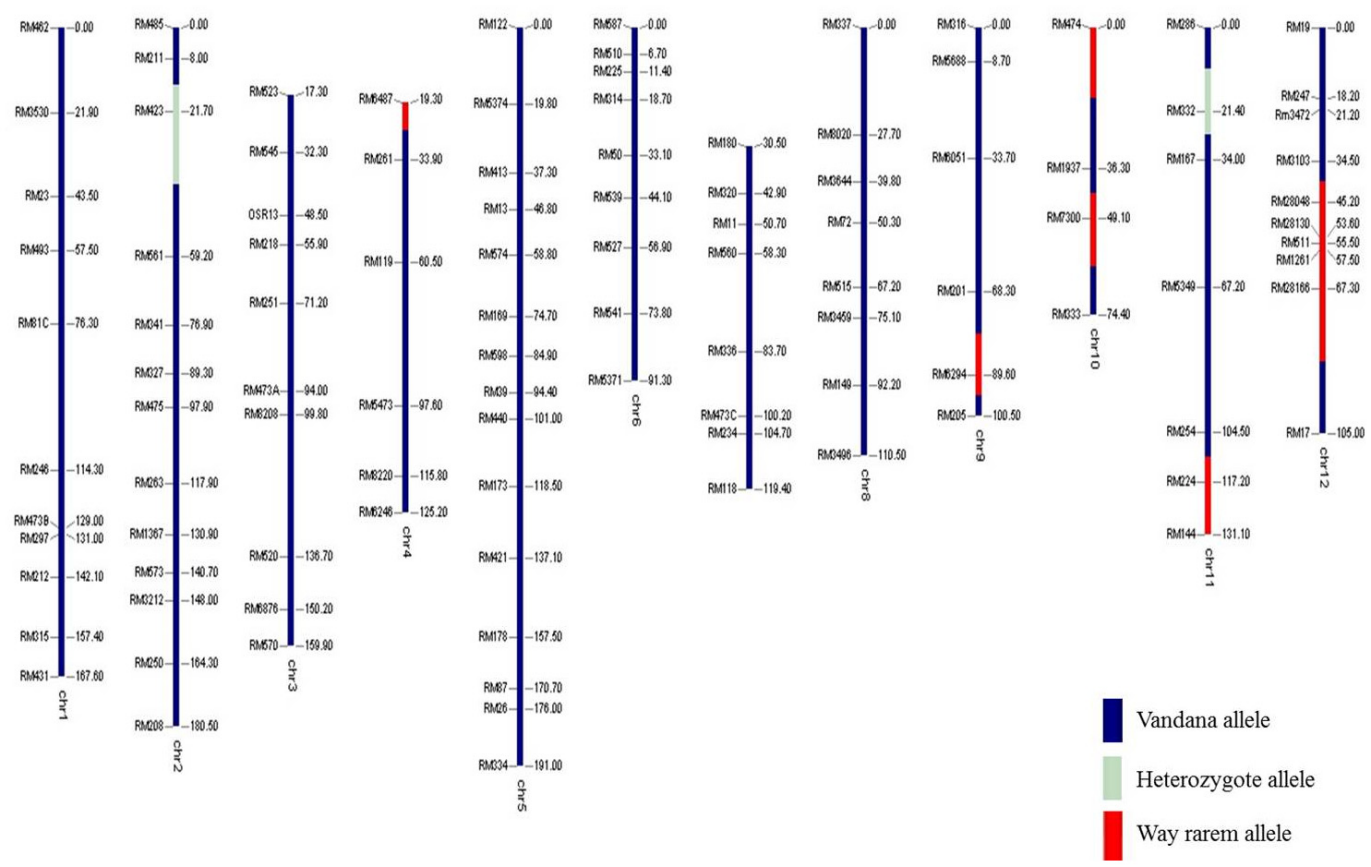
Genotypic background of *qDTY*_*12*.*1*_ BIL.

The performance of linkage-eliminated lines under RS is provided in Table 4. The *qDTY*_*3*.*1*_ Apo/Swarna BILs (IR88287-524-1-B-B and IR88287-63-1-B-B) showed significant GY increases under RS in two seasons of study. IR88287-524-1-B-B and IR88287-63-1-B-B, respectively, showed 344% and 386% increases over Swarna in the first season and 100% and 164% increases over Swarna in the second season under RS (Table 4). The *qDTY*_*12*.*1*_ lines IR84984-83-15-332-B and IR84984-83-15-817-B, respectively, showed GY enhancement of 349% and 109% over Vandana in the first experiment and 383% and 194% over Vandana in the second experiment under RS (Table 4). The dwarf *qDTY*_*1*.*1*_ N22/Swarna lines IR91659-41-59-B and IR91659-54-17-B showed a double cross-over event around *sd1* gene and significant GY increases of more than 300% in two experiments conducted in two different environments (Fig 2, Table 4). Genotypes developed through MAB recovered over 85% of the recipient parent genome (Figs 3, 4 and 5).

**Table 4:** Means (±SED) of QTL lines developed through marker-assisted breeding for high grain yield under drought.

QTL	Entry	Stress 1			Stress 2			Non-stress		
		DTF	PHT	GY	DTF	PHT	GY	DTF	PHT	GY
*qDTY*_*3*.*1*_	IR88287-524-1-B-B	108± 4*	61± 4*	1511± 360*	104± 1*	64± 3*	772± 202*	102± 4*	81± 4*	5358± 873^NS^
*qDTY*_*3*.*1*_	IR88287-63-1-B-B	104± 4*	62± 4*	1654± 360*	100± 1*	66± 3*	1018± 202*	96± 4*	73± 4*	4334± 873^NS^
	Swarna	109± 4*	62± 4*	340± 360*	109± 1*	62± 3*	385± 202*	103± 4*	82± 4*	4121± 873^NS^
*qDTY*_*12*.*1*_	IR84984-83-15-332-B	66± 3*	76± 4*	691± 175*	70± 3*	70± 4*	348± 73*	57± 1.4*	126± 4.6^NS^	3747± 744*
*qDTY*_*12*.*1*_	IR84984-83-15-817-B	64± 3*	71± 4*	322± 175*	72± 3*	61± 4*	212± 73*	56± 1.4*	118± 4.6^NS^	4130± 744*
	Vandana	69± 3*	66± 4*	154± 175*	70± 3*	64± 4*	72± 73*	54± 1.4*	120± 4.6^NS^	3556± 744*
*qDTY*_*1*.*1*_	IR91659:41-59-B	100± 3*	70± 5^NS^	2475± 645*	89± 3*	63± 3*	386± 28*	89± 11^NS^	97± 6^NS^	4428± 113^NS^
*qDTY*_*1*.*1*_	IR91659:54-17-B	93± 3*	75± 5^NS^	2440± 645*	90± 3*	71± 3*	383± 28*	82± 11^NS^	98± 6^NS^	4222± 113^NS^
	Swarna	98± 3*	75± 5^NS^	553± 645*	N	N	0	101± 11^NS^	91± 6^NS^	4778± 113^NS^

Stress 1 and Stress 2 correspond to the two different experiments. *qDTY*_*3*.*1*_ lines were screened in two different seasons whereas *qDTY*_*1*.*1*_ and *qDTY*_*12*.*1*_ lines were screened in only one season. *qDTY*_*1*.*1*_ lines were screened in two different environments (stress 1 = field conditions and stress 2 = rainout shelter). *qDTY*_*12*.*1*_ lines were screened on two different dates at the same location (stress 1 = first date and stress 2 = second date). Improved genotypes can be compared with their respective parents for each QTL (*qDTY*_*3*.*1*_, *qDTY*_*12*.*1*_ and *qDTY*_*1*.*1*_). DTF: days; PHT: cm, GY: kg ha^–1^. N: Swarna did not flower under a rainout shelter (severe stress).

*: Significance at 1%;

^NS^: Non-significant difference;

## Discussion

Molecular markers associated with drought resistance in plants offer a promise of increased selective gain to ensure sustainable targeted breeding progress [20]. Marker-assisted breeding using major drought GY QTLs in rice is a fast-track alternative approach to improve yield under drought [21,22]. Major drought GY QTLs that are potential candidates for an efficient drought molecular breeding program usually co-locate with other QTLs for traits such as DTF and PH, suggesting possible linkage or pleiotropy effects [9,12,13]. In addition, interactions of DTF and GY QTLs are highly likely because of the drought escape mechanism associated with DTF QTLs. Our study was conducted to test the pleiotropic effects of major drought GY QTLs, characterize interaction effects of drought GY and DTF QTLs, and finally define a molecular breeding strategy suitable for high GY under RS in rice.

The MT-MIM approach is being followed to test the significance of pleiotropic effects of the co-located QTLs in a particular chromosomal region [23,24]. The LOD values of pleiotropic effects obtained during the MT-MIM test with three drought GY QTLs were not significant (Table 1), clearly suggesting that there is no significant pleiotropic effect. Hence, there could be possible linkage between DTF/PH QTLs (or genes) and drought GY QTLs (or genes) co-locating in the same genomic region. Since the three drought GY QTLs (*qDTY*_*1*.*1*_, *qDTY*_*3*.*1*_, and *qDTY*_*12*.*1*_) analysed in our study co-located with QTLs for DTF under RS, it is worth looking into their effect under NS conditions. A QTL is considered as a flowering locus if it significantly affects DTF under both RS and NS conditions. Notably, *qDTY*_*1*.*1*_, *qDTY*_*3*.*1*_, and *qDTY*_*12*.*1*_ didn’t affect DTF under NS. Second, *qDTY*_*1*.*1*_ and *qDTY*_*3*.*1*_ significantly enhanced GY under RS even after covariate adjustment of DTF [9,12]. *qDTY*_*12*.*1*_ was identified in a subset of the population (242 lines out of 436) in which the effect of DTF was minimized [13]. Third, the MT-MIM test carried out in our study also supports possible linkage instead of pleiotropy.

Similar to DTF, the above-mentioned three drought GY QTLs co-located with PH QTLs under RS. Two of them (*qDTY*_*3*.*1*_ and *qDTY*_*12*.*1*_) were not associated with PH under NS, clearly indicating that the increase in PH was in response to drought. Second, these QTLs did not show a significant pleiotropic effect on GY and PH (Table 1). Unlike *qDTY*_*3*.*1*_ and *qDTY*_*12*.*1*_, *qDTY*_*1*.*1*_ was significantly associated with PH under NS. Contradictorily, in a CT9993/IR86626 population, *qDTY*_*1*.*1*_ showed a significant association with GY under RS but not with PH [7]. In three N22-derived RIL populations, Vikram et al. [25] have successfully proven that *qDTY*_*1*.*1*_, is linked with the *sd1* gene. The *qDTY*_*1*.*1*_ BILs developed in present study (IR91659:41-59-B and IR91659:54-17-B) had PH comparable with that of Swarna under both RS and NS conditions, suggesting a double crossover event at this locus (Table 4). Further analysis of these two lines with the *sd1* gene based marker confirmed the occurrence of double cross-over event (Fig 2).

In addition to linkages, genomic interactions play a crucial role in a drought MAB program. Digenic interactions have been reported to affect drought-related traits significantly [26]. Another recent example is the digenic interaction of a drought QTL on chromosome 3 (*qDTY*_*3*.*2*_) with another one on chromosome 12 (*qDTY*_*12*.*1*_) for GY under RS [15]. *qDTY*_*3*.*2*_ is a well-known flowering locus identified to show a digenic interaction with *qDTY*_*1*.*1*_ for DTF under RS in an N22/IR64 RIL population (Table 3). The digenic interaction of *qDTY*_*3*.*2*_ and *qDTY*_*1*.*1*_ was also observed in an N22/Swarna BIL population in DS2012. The N22 allele contributed to the reduction in DTF under RS in the two N22-derived populations. Interestingly, N22 alleles at *qDTY*_*3*.*2*_ and *qDTY*_*1*.*1*_ loci reduced DTF in the N22/Swarna RIL population, but no interaction was reported [9]. To better understand the effect of *qDTY*_*3*.*2*_ on two other drought GY QTLs, a QTL class analysis was carried out. The QTL class analysis of *qDTY*_*3*.*2*_, *qDTY*_*1*.*1*_, and *qDTY*_*12*.*1*_ revealed that *qDTY*_*3*.*2*_ interacts with other drought GY QTLs, resulting in decreased DTF under RS, consequently increasing GY under RS. On the other hand, GY under NS declines when DTY QTLs interact with *qDTY*_*3*.*2*_ (S2 and S3 Figs). To better understand the genetic effect of *qDTY*_*3*.*2*_ QTL, marker alleles at this locus was analyzed in an N22/Swarna BIL population and compared with results of a previous study on the N22/Swarna RIL population by Vikram et al. [9] where this QTL was first reported for high GY under RS. Reduction in DTF under RS and NS was due to the N22 allele. However, for GY under RS and NS, N22 and Swarna alleles had different effects. Under RS situation the N22 allele contributed for a GY enhancement whereas under NS situation Swarna allele contributed for the same. GY enhancement from the N22 allele under RS and from the Swarna allele under NS demonstrates that *qDTY*_*3*.*2*_ is a drought-responsive locus (Table 2, Fig 1). Another important note would be the effects of N22 and Swarna alleles on HI and BIO. The N22 allele increased HI and decreased BIO under both RS and NS situations. Similarly, Swarna allele decreased HI and increased BIO under RS as well as NS situations. GY increase under RS with the effect of N22 allele is likely to be due to mobilization of solutes from source to sink under RS. GY increase under NS with effect of the Swarna allele could be due the overall increase in BIO of plant. Drought-responsive and interacting loci such as *qDTY*_*3*.*2*_ should be pyramided with other desirable QTLs in a drought MAB program. Pyramiding can be followed for the development of relatively short-duration varieties (with minimum grain yield compromise), which are preferred for rainfed upland conditions.

**Fig 6:**
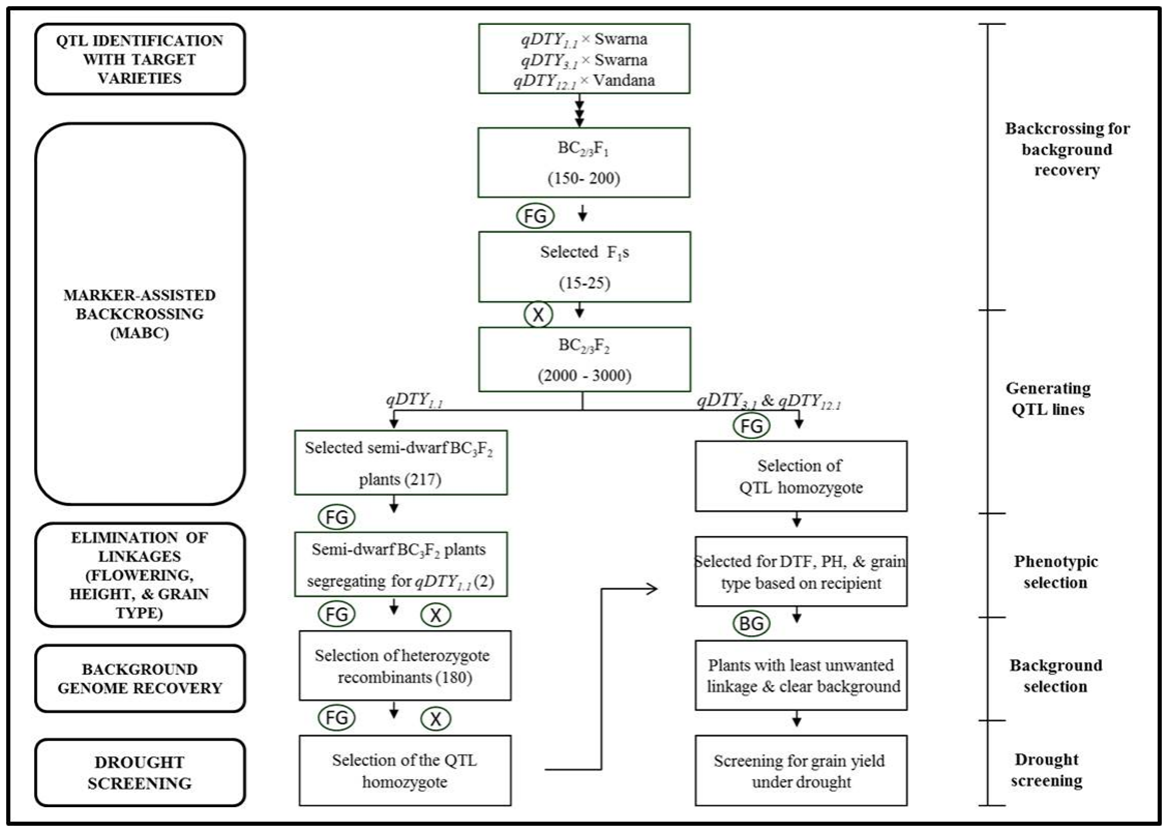
Figure presents the five important steps in marker-assisted breeding for high grain yield under drought in rice through major drought grain yield QTLs- *qDTY*_*1*.*1*_, *qDTY*_*12*.*1*_ and *qDTY*_*3*.*1*_. Figure also represents a separate strategy for elimination of linkage of the plant height gene from *qDTY*_*1*.*1*_ (*sd1* gene locus). FG = Genotyping with fore ground markers; BG = Genotyping for recipient background recovery.

The significant association of drought-related traits GY, PH, and DTF under RS in three important genomic regions (*qDTY*_*1*.*1*_, *qDTY*_*3*.*1*_, and *qDTY*_*12*.*1*_) is therefore due to their linkage effect, and this linkage need to be broken prior to their application in MAB. Practically, elimination of these linkages of PH and DTF from DTY QTLs could be carried out at the BC_n_F_2_ (n = 2/3) stage, that is, during selection of QTL homozygotes. There are background effects at the BC_n_F_2_ stage in addition to these linkages because of unidentified introgressions and unknown genomic interactions. A MAB strategy was followed to remove the effect of unwanted linkages, interactions and other background effects at the BC_n_F_2_ (n = 2/3) stage (Fig 6). Following this strategy dwarf *qDTY*_*1*.*1*_ Swarna lines with plant type and grain type similar to those of Swarna were developed showing more than a 300% yield advantage over Swarna under drought stress. Similarly, Swarna and Vandana introgression lines with *qDTY*_*3*.*1*_ and *qDTY*_*12*.*1*_ were also developed (Table 4).

Varying QTL effects in different backgrounds, unwanted linkages, and genomic interactions are the three most probable impediments in the deployment of drought QTLs in MAB programs. Drought GY QTLs offer promise of a selective advantage in drought MAB. The three major drought GY QTLs analyzed in our study through MT-MIM indicated possible linkages among PH, DTF, and GY genes/QTLs, which were eliminated during MAB. Interaction effects of DTF QTLs such as *qDTY*_*3*.*2*_ with *qDTY*_*1*.*1*_ and with *qDTY*_*12*.*1*_ in a previous study by Dixit et al. [15] suggested that these interacting loci also play a strategic role in a MAB program. It is advisable to use this information when defining a suitable MAB strategy for high GY under RS. Considering the linkage-interaction analysis of DTY QTLs in this study a suitable MAB protocol can be formulated with following steps: (1) QTLs for GY under RS could be identified in populations with target varieties in multiple populations simultaneously to identify major drought GY QTLs showing a significant effect across multiple, elite, and preferred genetic backgrounds [9]; (2) Co-locating QTLs could be tested for linkage vs pleiotropy for co-locating QTLs associated with phenotypically correlated traits; (3) Linkages of DTF and PH could be removed at the BC_2/3_F_2_ stage using a large population. In our study, linkages from the three drought QTLs (*qDTY*_*1*.*1*_, *qDTY*_*3*.*1*_, and *qDTY*_*12*.*1*_) were broken and drought-tolerant lines were developed; (4) The background of QTL homozygote lines could be tested genotypically in order to select lines with a higher representation of the recurrent parent genome; (5) Finally, screening of selected lines for GY under RS could be carried out to take advantage of other unidentified QTLs, genomic interactions, and/or irreversible epigenetic mechanisms (Fig 6). Such a modified MAB strategy would be advantageous in developing drought-tolerant rice varieties with a desired duration and increased grain yield under drought.

### Conclusion

Major and consistent drought GY QTLs *qDTY*_*1*.*1*_, *qDTY*_*3*.*1*_ and *qDTY*_*12*.*1*_ collocate with DTF and PH QTLs and this collocation is not due to pleiotropic effect, thereby indicating towards possible linkage. Allelic analysis of the *qDTY*_*3*.*2*_ revealed that it is a drought responsive locus. *qDTY*_*3*.*2*_ interacts *qDTY*_*1*.*1*_ and *qDTY*_*12*.*1*_ for reduction in flowering duration. A MAB strategy was followed for high grain yield under drought which involved elimination of the unwanted linkages and development of lines similar to recurrent parent. This MAB strategy could be followed for the development of drought tolerant lines with desired characters of recipient parents. Genetic improvement of the popular varieties which are susceptible to drought could be followed through this approach.

## Materials and Methods

Our study included a multiple-trait multiple-interval mapping (MT-MIM) analysis using phenotypic and genotypic data of populations in which *qDTY*_*1*.*1*_, *qDTY*_*3*.*1*_, and *qDTY*_*12*.*1*_ were originally identified [9,12,13]. Validation of the interacting locus, *qDTY*_*3*.*2*_, was carried out in N22-derived RILs and BILs. Further, epistatic interaction of *qDTY*_*3*.*2*_ with *qDTY*_*1*.*1*_ and *qDTY*_*12*.*1*_ was evaluated through a QTL class analysis to better understand the interaction of DTF and GY QTLs under RS and NS conditions. Finally, marker-assisted linkage elimination for all three QTLs was carried out.

### Mapping population data for testing pleiotropic effect of drought GY QTLs

Phenotypic and genotypic data of five mapping populations originally used for the QTL identification study were subjected to a test of pleiotropism. These populations were N22/Swarna, N22/IR64, Vandana/Way Rarem (RIL populations), and Apo/2*Swarna and IR74371-46-1-1/2*Sabitri (BIL populations). *qDTY*_*1*.*1*_ was identified in N22/Swarna and N22/IR64 [9], *qDTY*_*3*.*1*_ in Apo/2*Swarna [12], and *qDTY*_*12*.*1*_ in Vandana/Way Rarem [13] and IR74371-46-1-1/2*Sabitri [27].

### MT-MIM analysis of DTY-QTLs

A multiple-trait analysis model was used to test the pleiotropic effect of drought GY QTLs *qDTY*_*1*.*1*_, *qDTY*_*3*.*1*_ and *qDTY*_*12*.*1*_. This analysis was carried out through a Java-based QTL analysis software Q Gene version 4.3.10 [28]. The multiple-trait, multiple-interval mapping (MT-MIM) model used for analysis was similar to the multiple-trait composite interval mapping (MT-CIM) model explained by Kao et al. [29]. The only difference between these two models was that MT-MIM tests more than one QTL (denoted as ‘q’ in the formula) at a time, unlike MT-CIM, in which only one QTL is tested at a time [30]. The model used for MT-MIM was

$$\begin{array}{c} Y\\ n\;x\;t \end{array}=\;\sum \begin{array}{l} q\\ i\;=1 \end{array}\left(\begin{array}{cc} x_{i} & a_{i}\\ nx1 & 1xt \end{array}+\begin{array}{cc} z_{i} & d_{i}\\ nx1 & 1xt \end{array}\right)+\;\begin{array}{cc} X & B\\ n\;x\;\left(p+1\right) & \left(p+1\right)x\;t \end{array}+\;\begin{array}{c} E\\ n\;x\;t \end{array}$$ (1)

where 'q' is the number of QTLs being fitted simultaneously, t is the number of analyzed traits, 'n' is number of observation, 'p' is the number of non-genetic fixed factors, 'a_i_' and 'd_i_' are additive and dominant effects of a QTL, 'X' refers to the non-genetic fixed effect, 'B' is incidence matrix that links observation of the data with fixed effects and 'E' is random error Eq (1). The hypotheses to be tested are H0: ai = 0, di = 0 and E = 0 vs. HA: a ≠ 0, d ≠ 0 and E ≠ 0 (i.e., testing if the QTL effects are zero in at least one trait). In this case, there are multiple null hypotheses.

Co-locating GY QTLs were tested in pairs with either DTF or PH in the same QTL region at a time for LOD of pleiotropy effects with an assumption that there is linkage between the two if the LOD of the pleiotropy effects is not significant and there is pleiotropy if the LOD is significant. LOD thresholds at 5% and 1% significance levels were determined using 1000 permutations.

### Phenotyping

Phenotypic evaluation of two types of genetic material was performed in the study (1) N22/Swarna BIL population, and (2) QTL (*qDTY*_*1*.*1*_, *qDTY*_*3*.*1*_ and *qDTY*_*12*.*1*_) homozygote lines. Screening of the N22/Swarna BIL population of 297 lines was carried out under lowland RS and NS conditions in DS2011 and DS2012. Experiments were laid out in an alpha lattice design in two replications with a 5-meter (m) single-row plot and 0.2-m row spacing. Seeds were sown in a nursery and 21-day-old seedlings were transplanted with single seedlings per hill. Nitrogen, phosphorus, and potassium (NPK) were applied at 120:30:30 kg ha^-1^. Bayluscide (niclosamide, 0.25 kg a.i. ha^-1^) was sprayed immediately after transplanting for snail control. For weed control, Sofit (pretilachlor + safener, 0.3 kg a.i. ha^-1^), a post-emergence herbicide, was sprayed 4 days after transplanting (DAT). Furadan (carbofuran, 1 kg a.i. ha^-1^) and Cymbush (cypermethrin, 1 L ha^-1^) ± Dimotrin (cartap hydrochloride, 0.25 kg a.i. ha^-1^) were applied at 5 DAT and 16 DAT, respectively, for insect pest control.

A standing water level of 5 cm was maintained after transplanting throughout the crop season and drained before harvesting in the NS experiments. The RS experiments were maintained similar to the NS experiments until 30 DAT and water was drained for the imposition of stress at 30 DAT. The RS experiments were re-watered after 70% of the population lines showed severe leaf rolling symptoms. After 24 hours, the fields were drained again for a second RS cycle [31].

The phenotypic data for GY, DTF, and PH were recorded in DS2011. Further, two more parameters, biomass (BIO) and harvest index (HI), were added in the DS2012 experiment. When panicles of 50% of the plants in each plot were exerted, DTF was recorded. PH (cm) was measured as the height from the soil surface to the tip of the panicle on the main tiller before harvesting. BIO (g m^-2^ converted to kg ha^-1^) was taken from a 1-m^2^ area in each plot. Harvested samples were oven-dried, weighed, and threshed for grain weight. Grains were dried to 12% moisture before weighing [12, 13].

To calculate HI, the following formula Eq (2) was used:

$$\text{Harvest index}=\frac{\text{Grain weight}}{\text{Total biomass weight}}$$ (2)

QTL homozygote lines were screened in two different experiments (1) Stress 1 and Stress 2. *qDTY*_*3*.*1*_ lines were screened in two different seasons. *qDTY*_*1*.*1*_ lines were screened in two different environments (stress 1 = field conditions and stress 2 = rainout shelter). *qDTY*_*12*.*1*_ lines were screened on two different dates at the same location (stress 1 = first date and stress 2 = second date). Phenotypic data of an N22/IR64 population previously published by Vikram et al. (2011) were also used for the *qDTY*_*3*.*2*_ validation and interaction study.

### Genotyping

Similar to the phenotyping, genotyping was also carried with (1) N22 derived BILs and RILs and (2) QTL homozygote lines. Genotyping of the N22/3*Swarna BIL population used for the *qDTY*_*3*.*2*_ validation in our study was carried out. In addition to this, an N22/IR64 RIL population was genotyped with markers for the *qDTY*_*3*.*2*_ QTL region to analyze its interaction effects with *qDTY*_*1*.*1*_. The N22/IR64 RIL population was originally used for the identification of *qDTY*_*1*.*1*_ [9].

Freeze-dried leaf samples were cut in eppendorf tubes, ground using a GENO grinder, and DNA was extracted following a modified CTAB method [32]. Quantification was done on a 0.8% agarose gel and concentrations adjusted to ~25 ng μL^-1^ for the PCR amplification. A 15-μL PCR mixture contained 50 ng DNA, 1 × PCR buffer, 100 μM dNTPs, 250 μM primers, and 1 unit *Taq* polymerase enzyme. PCR products were resolved in 8% non-denaturing polyacrylamide gel electrophoresis (PAGE). Genotyping of both N22/Swarna BIL and N22/IR64 RIL populations was done with markers for *qDTY*_*1*.*1*_ and *qDTY*_*3*.*2*_ (RM212, RM315, RM3825, RM11943, RM431, RM12023, RM12091, RM12146, RM22, and RM231).

QTL homozygote lines for three QTLs *qDTY*_*1*.*1*_, *qDTY*_*3*.*1*_ and *qDTY*_*12*.*1*_ were genotyped for their respective foreground markers reported by Vikram et al. [9], Venuprasad et al. [12] and Bernier et al. [13]. Marker analysis was also done for determining background recovery. A polymorphism survey was performed and markers spaced at equal distance throughout genome were selected for testing genetic background (Figs 3, 4 and 5).

### Statistical and QTL analysis

Statistical analysis in the N22/Swarna BIL population was done using CROPSTAT v.4.2.3 (available at www.irri.org). Phenotypic means of entries were estimated using the following linear mixed model for the analysis of variance:

$${\text{P}}_{\text{ijk}}{=\text{M}+\text{R}}_{\text{i}}{+\text{B}}_{\text{j}}\left({\text{R}}_{\text{i}}\right){\;+\text{L}}_{\text{k}}{+\text{e}}_{\text{ijk}}$$ (3)

where P_ijk_ is the measurement recorded on a plot, M is the mean over all plots, and R, B, L, and e are replications, blocks, lines, and error, respectively and i, j and k are number of replicates, blocks and lines respectively Eq (3). For estimating the entry means, replications and blocks within replicates were considered random whereas entries were fixed.

Genetic map distances between markers were estimated using their physical distances. Genetic distances between markers were estimated using Map Manager QTX software [33]. LOD threshold of 3.0, significance value of 1% (P ≤ 0.01) and Kosambi map function was used for the estimation of genetic distances. QTL analysis was with QTL Network software v2.1 using a mixed model-based composite interval mapping method [34] in which the significance level of the marker intervals was tested for their associations with traits of interest. Candidate marker intervals were selected and used as co-factors in a one-dimensional genome scan. Determination of the candidate intervals, detection of putative QTLs, and their additive effects were significant at P < 0.01. The F-value threshold was determined using 1000 permutation tests. Window size and walk speed used for the genome scan were 10 cM and 1 cM, respectively.

### QTL class analysis for *qDTY*_*3*.*2*_ interaction analysis

Digenic interactions between DTF and PH QTLs in *qDTY*_*1*.*1*_ and *qDTY*_*3*.*2*_ regions were identified. To better understand the interaction of drought GY and DTF QTLs, QTL classes were formed. QTL classes were *qDTY*_*1*.*1*_, *qDTY*_*3*.*2*_, *qDTY*_*12*.*1*_, *qDTY*_*1*.*1*_ + *qDTY*_*3*.*2*_, and *qDTY*_*12*.*1*_ + *qDTY*_*3*.*2*_. Therefore there were total of four classes- (1) Class I with *qDTY*_*12*.*1*_ or *qDTY*_1.1,_ (2) Class II with *qDTY*_*3*.*2*,_ (3) Class III with *qDTY*_*3*.*2*_ + qDTY_*12*.*1*_ / *qDTY*_*1*.*1*_ and (4) Class IV with no QTL. Number of lines under class I, II, III and IV in N22/IR64 population were-17, 6, 12 and 4 respectively, whereas class I, II, III and IV had- 18, 7, 11 and 4 genotypes in N22/Swarna population respectively. In Vandana/ WayRarem population class I, III and IV had- 27, 2 and 30 genotypes respectively. None of genotype with only *qDTY*_*3*.*2*_ was found. However, one genotype with this QTL was taken in which background QTLs were in segregating condition. Phenotypic means of the lines with and without QTLs and QTL combinations were estimated. QTL class analysis was carried out with phenotypic data of RS as well as NS experiments.

### Marker-assisted linkage elimination

A marker-assisted breeding strategy was followed for the elimination of linkage of PH from DTY QTL *qDTY*_*1*.*1*_. The marker-assisted linkage elimination was done to remove the linkage of PH and DTF QTLs from *qDTY*_*1*.*1*_, *qDTY*_*3*.*1*_, and *qDTY*_*12*.*1*_. QTL lines for the three QTLs (*qDTY*_*1*.*1*_, *qDTY*_*3*.*1*_, and *qDTY*_*12*.*1*_) were backcrossed with their respective recipient parents to recover the recipient genotype background. *qDTY*_*1*.*1*_ and *qDTY*_*3*.*1*_ lines were backcrossed thrice whereas *qDTY*_*12*.*1*_ lines were backcrossed twice. The total number of F_1_s produced ranged from 150 to 200 in the three crosses. These F_1_s were genotyped with foreground markers and 15–25 lines segregating for QTLs were selected. Selected BC_2_F_1_s were selfed to generate 2000–3000 BC_n_F_2_ (BC_n_F_2_ refers to BC_2_F_2_ or BC_3_F_2_) plants that were further genotyped for foreground markers of the respective QTLs, and finally QTL homozygote lines were selected. This strategy was followed for *qDTY*_*3*.*1*_ and *qDTY*_*12*.*1*_ to remove PH and DTF QTL linkages. A slightly different strategy was followed for *qDTY*_*1*.*1*_ as described by Vikram et al [25]. Semi-dwarf plants were selected among BC_3_F_2_ plants and genotyped for *qDTY*_*1*.*1*_ foreground markers. Plants segregating for *qDTY*_*1*.*1*_ were allowed to segregate twice more to generate the QTL homozygote. Further selection among QTL homozygote lines was made for DTF and PH. Background genotyping of the QTL homozygote was carried out (Fig 3). Similarly, *qDTY*_*3*.*1*_ and *qDTY*_*12*.*1*_ BILs were genotyped to clear the background (Figs 4 and 5). Finally, lines with cleared backgrounds and recurrent parent phenology were screened for GY under RS. *qDTY*_*1*.*1*_ BIL lines were screened in lowland RS conditions in DS2012 at two locations: RS in the field (stress 1) and in a rainout shelter (stress 2). The *qDTY*_*3*.*1*_ BIL lines were screened under lowland RS in DS2011 (stress 1) and DS2012 (stress 2). The screening protocol used for phenotyping of the N22/Swarna BILs was followed. The *qDTY*_*12*.*1*_ BILs were screened under upland RS in DS2010 in two different experiments sown at two different dates (first date: stress 1 and second date: stress 2) using a protocol described by Bernier et al. [13].

## Supporting Information

S1 Fig Epistatic interaction of *qDTY*_*1*.*1*_ and *qDTY*_*3*.*2*_ for days to 50% flowering in an N22/IR64 RIL population. The additive interaction of *qDTY*_*3*.*2*_ with *qDTY*_*1*.*1*_ reduced days to 50% flowering under stress as well as non-stress situations.
(TIF)

S2 Fig QTL class analysis: interaction of flowering QTL (*qDTY*_*3*.*2*_) with grain yield QTLs (*qDTY*_*1*.*1*_ and *qDTY*_*12*.*1*_) under drought stress. Trait values (DTF or GY) and QTLs/ QTL combinations have been plotted on ‘Y’ and ‘X’ axis respectively. Trait values (DTF or GY) of QTL combination class circled with orange colour to show that how *qDTY*_*3*.*2*_ interacts with *qDTY*_*1*.*1*_ and *qDTY*_*12*.*1*_ to reduce flowering duration and enhance grain yield under drought in different populations. Standard errors of difference for different classes of three populations were (1) N22/IR64 RIL population: DTF = 1.589, GY = 232.1, (2) N22/Swarna: DTF = 2.409, GY = 256.3; (3) Vandana/Way Rarem: DTF = 3.415, GY = 128.2.
(TIF)

S3 Fig QTL class analysis: interaction of flowering QTL (*qDTY*_*3*.*2*_) with grain yield QTLs (*qDTY*_*1*.*1*_ and *qDTY*_*12*.*1*_) under non-stress. Trait values (DTF or GY) and QTLs/ QTL combinations have been plotted on ‘Y’ and ‘X’ axis respectively. Trait values (DTF or GY) of QTL combination class (*qDTY*_*3*.*2*_ and *qDTY*_*1*.*1*_ */ qDTY*_*12*.*1*_) circled with orange colour. Lines with *qDTY*_*1*.*1*_ and *qDTY*_*3*.*2*_ had lower yield as compared to the lines with only *qDTY*_*1*.*1*_. On the other hand *qDTY*_*12*.*1*_ and *qDTY*_*3*.*2*_ had lower grain yields compared to the lines with either *qDTY*_*12*.*1*_ or *qDTY*_*3*.*2*_. Standard errors of difference for different classes of three populations were (1) N22/IR64 RIL population: DTF = 2.010, GY = 502.7, (2) N22/Swarna: DTF = 1.754, GY = 497.8; (3) Vandana/Way Rarem: DTF = 3.913, GY = 304.9.
(TIF)

## Abbreviations

BIL: Backcross inbred line
BIO: Biomass
CTAB: Cetyl trimethyl ammonium bromide
DNA: Deoxyribonucleic acid
DTF: Days to 50% flowering
GY: Grain yield
HI: Harvest index
LOD: Logarithm of odds
MAB: Marker-assisted breeding
NS: Non-stress
PCR: Polymerase chain reaction
PAGE: Polyacrylamide gel electrophoresis
PHT: Plant height
QTL: Quantitative trait loci
RS: Reproductive-stage drought stress
