# Supplementary figures and images for: Linkages and Interactions Analysis of Major Effect Drought Grain Yield QTLs in Rice
Source: PLoS One. 2016 Mar 28;11(3):e0151532. doi: 10.1371/journal.pone.0151532 (PMC4809569; doi:10.1371/journal.pone.0151532)

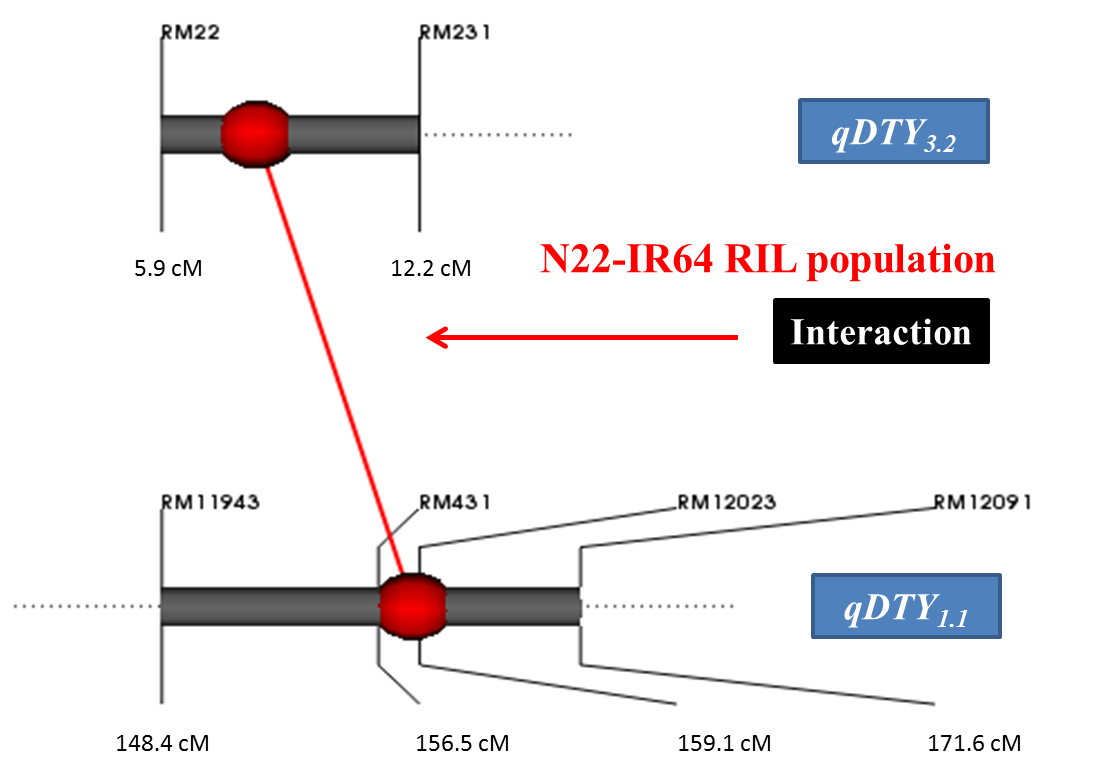

Supplement: S1 Fig — The additive interaction of qDTY3.2 with qDTY1.1 reduced days to 50% flowering under stress as well as non-stress situations. (TIF) [file pone.0151532.s001.tif]

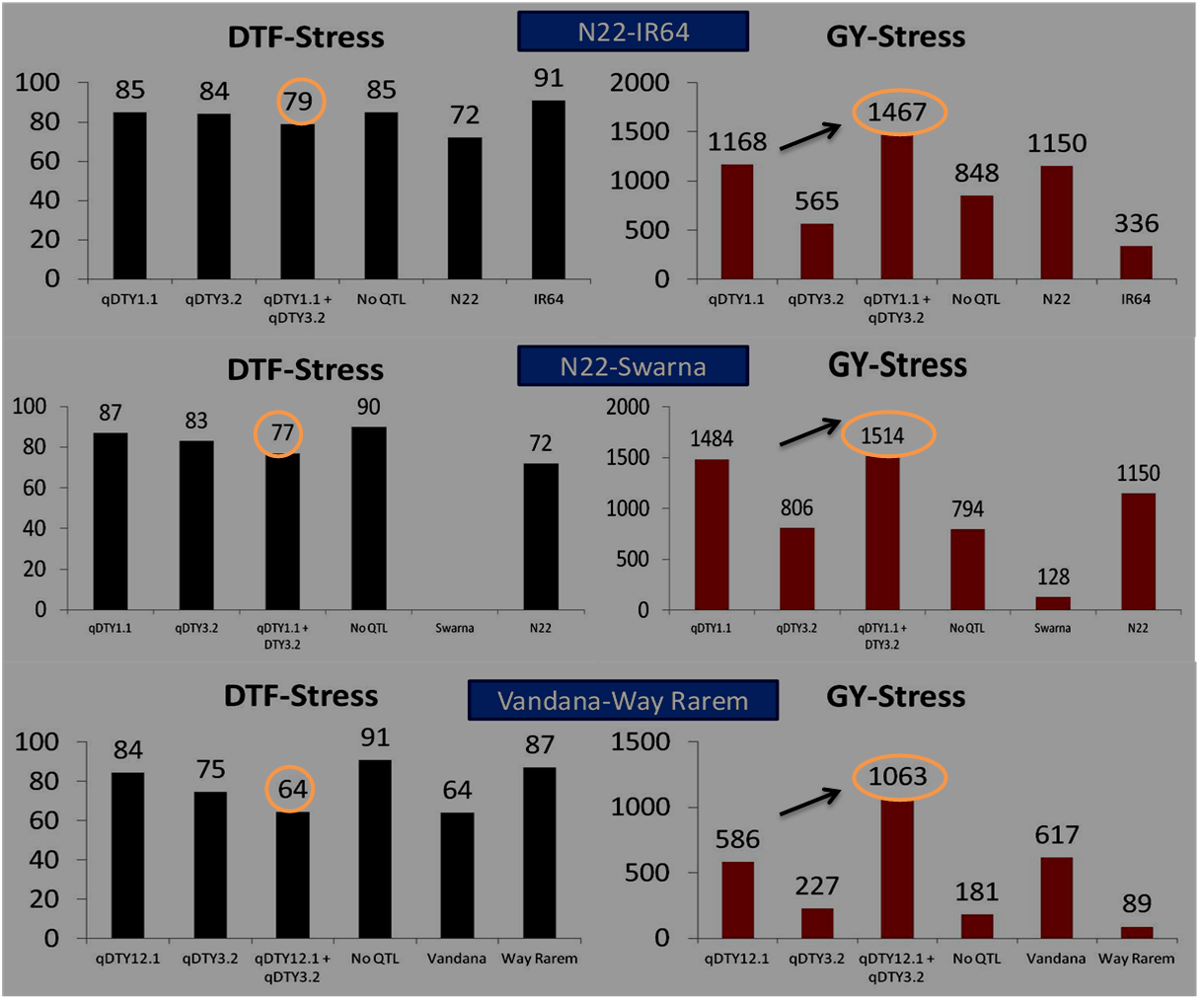

Supplement: S2 Fig — Trait values (DTF or GY) and QTLs/ QTL combinations have been plotted on ‘Y’ and ‘X’ axis respectively. Trait values (DTF or GY) of QTL combination class circled with orange colour to show that how qDTY3.2 interacts with qDTY1.1 and qDTY12.1 to reduce flowering duration and enhance grain yield under drought in different populations. Standard errors of difference for different classes of three populations were (1) N22/IR64 RIL population: DTF = 1.589, GY = 232.1, (2) N22/Swarna: DTF = 2.409, GY = 256.3; (3) Vandana/Way Rarem: DTF = 3.415, GY = 128.2. (TIF) [file pone.0151532.s002.tif]

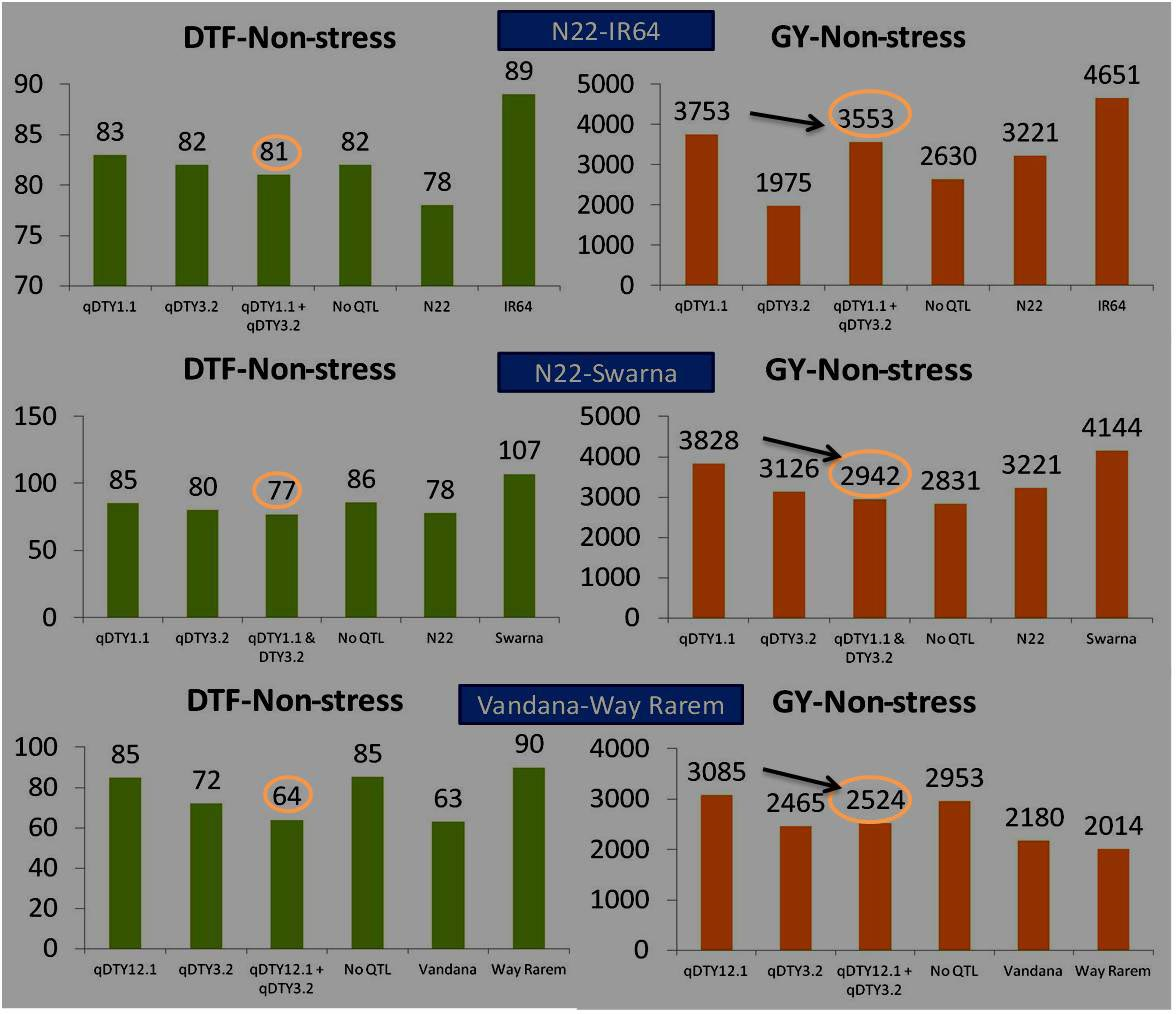

Supplement: S3 Fig — Trait values (DTF or GY) and QTLs/ QTL combinations have been plotted on ‘Y’ and ‘X’ axis respectively. Trait values (DTF or GY) of QTL combination class (qDTY3.2 and qDTY1.1 / qDTY12.1) circled with orange colour. Lines with qDTY1.1 and qDTY3.2 had lower yield as compared to the lines with only qDTY1.1. On the other hand qDTY12.1 and qDTY3.2 had lower grain yields compared to the lines with either qDTY12.1 or qDTY3.2. Standard errors of difference for different classes of three populations were (1) N22/IR64 RIL population: DTF = 2.010, GY = 502.7, (2) N22/Swarna: DTF = 1.754, GY = 497.8; (3) Vandana/Way Rarem: DTF = 3.913, GY = 304.9. (TIF) [file pone.0151532.s003.tif]
